# Protection against neonatal enteric colibacillosis employing *E. Coli*-derived outer membrane vesicles in formulation and without vitamin D3

**DOI:** 10.1186/s13104-018-3442-2

**Published:** 2018-05-16

**Authors:** Babak Beikzadeh, Gholamreza Nikbakht Brujeni

**Affiliations:** Department of Microbiology and Immunology, Faculty of Veterinary Medicine, University of Tehran, Azadi Avenue, Tehran, Iran

**Keywords:** Enterotoxigenic *Escherichia coli*, Outer membrane vesicles, Vitamin D3

## Abstract

**Objective:**

Enterotoxigenic *Escherichia Coli* (ETEC) is the cause of diarrhea and even death in humans and offspring of animals. Outer membrane vesicles (OMVs) of the ETEC was prepared and its potential as a vaccine candidate against enteric colibacillosis in neonatal mice was evaluated. Dam mice intradermally injected with ETEC-derived OMVs and OMVs plus an active form of vitamin D3 (avD3). Mucosal and systemic immune responses in mice and passive immunity protection against ETEC lethality in their offspring was investigated.

**Results:**

Immunization of adult mice via ETEC-derived OMV alone and in formulation with avD3 protect offspring from ETEC-induced lethality. Nevertheless, avD3 did not indicate a positive effect on mucosal and systemic immune responses. Only the combination of OMV plus avD3 elicited a significant (*P *< 0.05) increase in the level of specific IgA antibodies in serum.

## Introduction

Outer membrane vesicles (OMVs) are a non-living bacterial part that has been used as vaccine candidates [1]. They are secreted from the cell surface and possess proteins, lipopolysaccharides, phospholipids, periplasmic components, DNA and RNA. Using OMVs for immunization purposes have some advantages over common vaccines such as cost–benefit and availability, having partially or whole the virulence factors, and do not require an adjuvant [2–5].

Enterotoxigenic *E. coli* (ETEC) is an important cause of lethal diarrhea in neonatal calves (Colibacillosis), piglets and sometimes in humans [6–11]. Active immunization of neonates against disease is not practicable and passive immunity is necessary to protect during the first days of life [12]. Despite the massive works done on vaccine inoculation design to prevent the infection in mothers and offspring’s, no broadly protective vaccine is now available, especially for newborn animals [13–15]. Studies on ETEC-derived OMVs have shown that immunization with these particles leads to produce antibodies against bacteria [11, 15–18]. These results demonstrate the active immune responses against OMVs, but they do not show, if the neonate is infected after 24 h of birth, whether the maternal derived antibodies could protect them from bacteria-induced lethality.

Although there are several whole germ-attenuated, killed or recombinant vaccines to prevent the disease [14, 19–21], the bacteria may still lead to the infection of neonates in the early hours after birth [22, 23]. As OMVs stimulates the systemic immune response and avD3 can stimulates the mucosal immunity [24–26], we hypothesized that applying avD3 along with OMVs can switch systemic immune responses to mucosal response and robust mucosal immunity and finally increase protection against ETEC in neonatal mice.

## Main text

### Materials and methods

#### OMV isolation and characterization

Bovine ETEC O101: K99 (field strain) were cultured in Luria–Bertani (LB) broth (Merck, Germany) with aeration or, if necessary, LB broth agar plate at 37 °C. Isolation of OMVs was performed as described previously [27]. Bacterial culture was pelleted at 10,000×*g* for 15 min and then the supernatant was transferred to Tangential Flow Filtration system (TFF) (Millipore, DUOBLOC TM, USA) to concentrate high molecular weight proteins and remove low molecular weight proteins (10,000-molecularweight-cutoff). OMVs were prepared with extra filtration through 0.45 and 0.22 µm filters. Finally, the supernatant was pelleted using a high-speed centrifuge (Refrigerated SIGMA 3-16K Centrifuge) at 20,000×*g* for 3 h at 4 °C. Isolated OMVs were aliquoted in PBS and sorted at − 80 °C.

Transmission electron microscopy [11] was used to verify OMV morphology based on Park et al. with some modifications [18]. Vesicles were resuspended in 0.01 M PBS and then passed through a nickel grade 400 mesh. Next10 µl of OMV sample was placed on coated grade with the carbon-reinforced formvar film. After 30 min at room temperature, the grade was washed with 0.01 M PBS solution (0.5 M BSA and %0.1 gelatin). The grid was fixed with 0.01 M PBS containing 1% glutaraldehyde at 4 °C for 1 h and then washed again with 0.01 M PBS. Finally, the grid was stained with 2% phosphotungstic acid (negative staining). Finally, images were obtained using microscope software ZEN lite from ZEISS EM900 transmission electron microscopy.

#### Immunization regime and challenge protocol

The source of animals and experiment procedures were approved and monitored by animal care center, Faculty of Veterinary Medicine, University of Tehran. The study population consists of 30 female mice (BALB/c background, 6 weeks old) divided into three groups, containing 10 mice in each group (n = 5 mice as sham and n = 5 mice as immunized). Each two female mice were mated with one age-matched male and immunization was started at day 0, 14 and 28 with OMV alone (two groups) and OMV plus avD3 (one group) via i.d. route following this concentration: for OMV, 100 µg [28, 29] and for avD3 (1α,25-Dihydroxycholecalciferol- Sigma-D1530), 0.1 µg of avD3 in 0.2 µl of 95% of ethanol [19, 25] was add to each dose of vaccine. After the pregnancy, the dam mice were separated and monitored until birth. After 24 h of suckling, all neonatal mice were subjected to oral challenge with 10^2^ and 10^3^ CFU of ETEC [30] and returned to their dams to allow a continuous transferring of immunoglobulin from dams to infected offspring. The survival rate of neonatal was recorded for 7 days.

#### Collection of samples

Newborn mice at day 7 after challenge and their mothers at week 8 after immunization were euthanized and the blood collected by cardiac puncture. Since all neonates from sham group died in the first 24 h after challenge, another unchallenged group were sampled. To obtain intestinal lavage fluids, the intestine samples were washed three times with ice-cold PBS containing protease inhibitors. Samples were centrifuged 2500×*g* for 20 min at 4 °C and the supernatants were sorted at − 20 °C.

#### Measurement of antibodies titer against OMV

Serum and mucosal IgG and IgA titers were determined by an enzyme-link immunosorbent assay (ELISA) as described by Schild et al. [31].

### Immunization effect on ETEC removal

To confirm that recovered bacteria from the intestine were the challenge strain, PCR amplification procedure was performed according to Franck et al. [32].

#### Identification of immunogenic proteins

Prepared OMVs and two ETEC strains including field isolated strain, O101:K99, and a reference ETEC strain 510, along with the recombinant K99 were separated by sodium dodecyl sulfate–polyacrylamide gel electrophoresis (SDS-PAGE) and the specificity of OMV-derived antibodies were tested by western blot analysis as previously described [33].

### Statistical analysis

Survival curves were analyzed using Log-rank (Mantel-Cox) test. Student’s t test and the One-way ANOVA (Tukey’s multiple comparisons test) were used to assess significant differences between groups. All values were expressed as mean ± SEM.

## Results

### ETEC-derived OMVs immunization and survival rate of neonatal

**Fig. 1:**
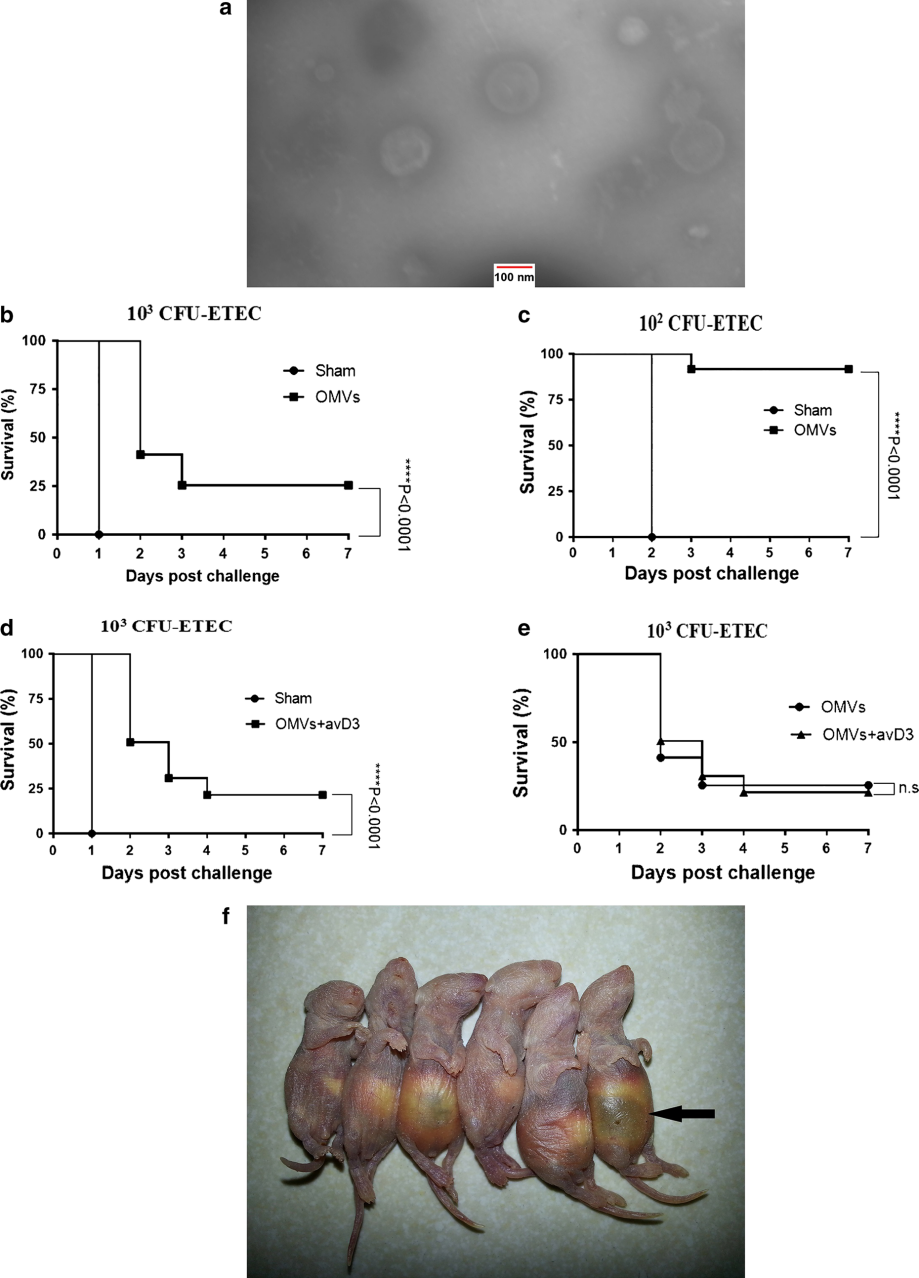
**a** Electron micrograph of ETEC O101:K99—derived OMVs. To confirm final product purification of ETEC culture supernatant and vesicles morphology, they were negatively stained and visualize by Transmission electron microscopy. Image was obtained by ZEISS EM900. Bar = 100 nm. Survival rates of respective offspring from immunized and sham-immunized mice. To evaluated the effect of OMV-and sham (PBS) immunization regime on neonatal mice protect against ETEC, **b** suckling offspring (n = 50–53, each group) after 24 h of birth challenged orally with lethal doses of ETEC (10^3^ CFU) and survival was monitored every 12 h for 7 days. **c** suckling offspring (n = 50–53, each group) after 24 h of birth challenged orally with lethal doses of ETEC (10^2^ CFU) and survival was monitored every 12 h for 7 days **d** Likewise, respective suckling offspring (n = 52–63, each group) from OMVs plus avD3-and sham (PBS + avD3) immunization mice, after 24 h of birth challenged orally with 10^3^ CFU. All sham-immunized mice died in the first 24 and 48 h after challenged with 10^3^ and 10^2^ CFU of bacteria respectively. **e** Survival rate comparison between OMV-immunized group and OMV plus avD3-immunized group. No significant difference was observed in survival rate due to use avD3. **f** Ascites was seen as post mortem symptom in all neonatal mice that had no ability to resist against disease (black arrow)

The OMVs, which were obtained from ETEC, have shown spherical shape in different size (50–100 nm), without cell debris (Fig. 1a). After morphological characterization and determination of protein concentration, mice were immunized by ETEC-derived OMVs in formulation with avD3. All sham-immunized group quickly died at first 24 h of post-challenge, while 25.49% of OMV immunized group survived until day 7 (Fig. 1b). In contrast, the survival rate of neonatal mice challenged with a low dose of ETEC (10^2^ CFU) was significantly improved to 91.66% (p < 0.0001) (Fig. 1c). The OMV plus avD3 group and sham-immunized group (PBS + avD3) indicated a similar pattern with OMV group. Only 21.3% of immunized group survived at day 7 (p < 0.0001) (Fig. 1d, e). As a result of bacterial pathogenesis in the sham-immunized group, ascites (accumulation of fluid in the abdomen) was seen in the neonatal mice (Fig. 1f). Survived neonate from immunized mice did not show any adverse physical symptom. Overall, these results demonstrate that immunization of adult mice against ETEC via ETEC-derived OMV protects offspring from ETEC-induced lethality, even in formulation with avD3.

### Systemic versus mucosal antibody response

Sera from immunized group- mother mice (MM) with OMV as well as OMV plus avD3 group indicated a significant increase in IgG level compared to sham-immunized mice. Group immunized by solely OMVs produced more IgG titer than other groups. The levels of IgA titer revealed that only the combination of OMV plus avD3 has the potential to produce IgA antibodies in serum. However, in the intestine, there was no significant difference in IgA level between immunized and sham-immunized group.

**Fig. 2:**
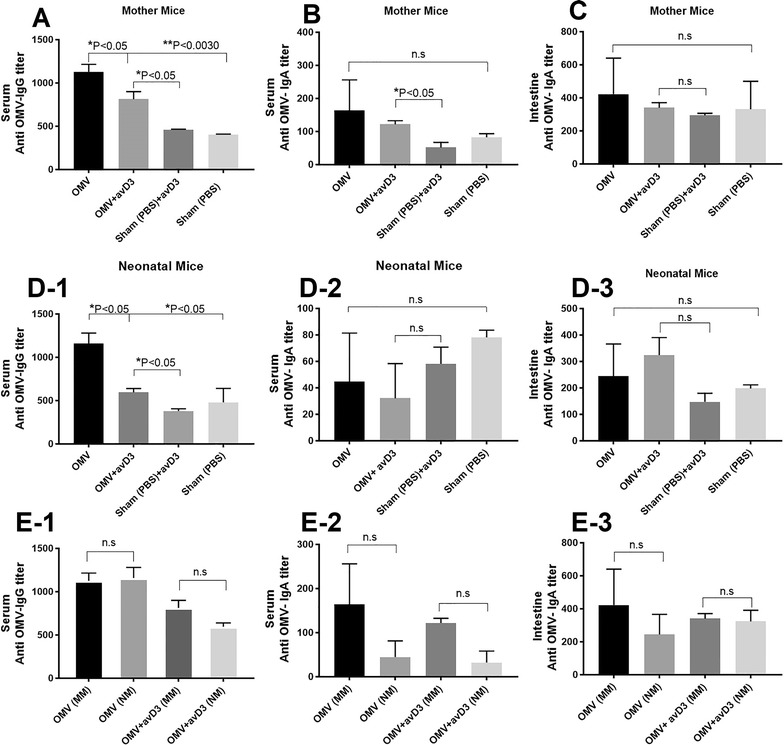
Serum and intestinal anti OMV- IgG and IgA titers. **a**–**c** Mother mice (n = 5, each group) serum IgG and IgA titers at week 8 after immunization and intestinal IgA titer. (D1–3) Respective offspring (n = 15–18, each group) IgG and IgA titers at day 7 after challenge. (E1–3) Comparison of IgG and IgA titers in serum and intestine of mother mice and neonatal mice (NM). Comparison of all groups were performed by One-way ANOVA- Tukey’s multiple comparison test. Bars represented pooled results (geometric mean ± SEM)

In concordance with MM antibodies response, offspring had a similar level of IgG in their serum and IgA in their intestine. To evaluate whether maternal antibodies are transferred to neonate or not, the levels of IgG and IgA of MM were compared to those of neonatal mice (NM). Interestingly, the results showed that the levels of antibodies in NM are directly correlated with MM (Fig. 2). However, OMVs had the capability to induce a systemic antibody response, while the avD3 had no effect on mucosal response.

### ETEC removal from intestine

ETEC K99 positive bacteria was detected in all neonates from immunized and sham-immunized groups that were challenged with 10^3^ CFU. Only the challenged neonates with a 10^2^ CFU were negative.

### Specificity of antibody response

**Fig. 3:**
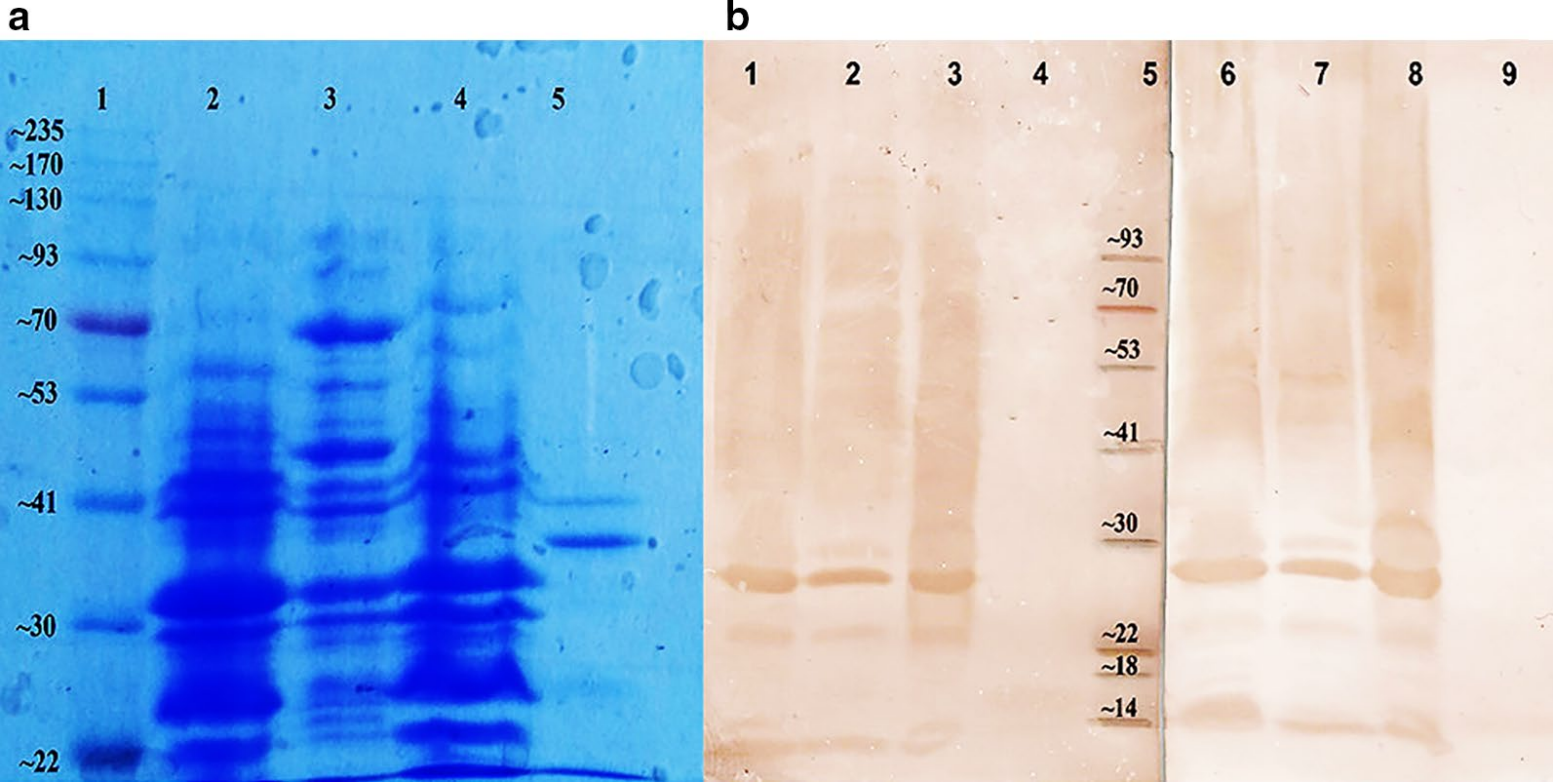
Specificity of IgG response from immunized and sham-immunized mice. **a** SDS-PAGE gel: molecular mass of protein standard in kDa (lane 1), ETEC O101:K99 strain (lane 2), OMVs (lane 3), ETEC 510 (O101:K99) strain (lane 4) and purify K99 protein (lane 5). **b** Immunoblot of SDS-PAGE gel using immunized sera: ETEC O101:K99 strain (lane 1), OMVs (lane 2), ETEC 510 (O101:K99) strain (lane 3), purify K99 protein (lane 4), molecular mass of protein standard in kDa (lane 5). Immunoblot of SDS-PAGE gel using sham-immunized sera: ETEC O101:K99 strain (lane 6), OMVs (lane 7), ETEC 510 (O101:K99) strain (lane 8) and purify K99 protein (lane 9)

Western blotting analysis showed specific antibodies that react with multiple bands in OMV extract and whole germ bacteria. Our results indicated the presence of immune dominant proteins (~ 14, ~ 22 and ~ 30 kDa) in OMVs and whole germ bacteria. Comparing to the whole germ ETEC O101: K99^+^and OMVs, no reactivity in the serum of immunized mice was found for purified K99 peptide (Fig. 3).

## Discussion

Although efforts have been performed in the field of protective colibacillosis vaccine [6, 17], a broadly effective vaccine has not been produced yet. In this work, the potential of ETEC-derived OMVs plus avD3 in the induction of mucosal immunity and increase protection against ETEC in the neonate was investigated. ETEC vesicles were isolated from concentrated supernatant through several steps of filtration. Isolated OMVs in terms of morphology such as the range size and purity were confirmed with the others reports [11, 15, 16, 18].

To evaluate the immunogenicity of OMVs and the effects of avD3, we choosed the i.d. route for stimulating the Langerhans cells and induction a strong immune response [34–38]. As expected, OMV immunized group has a higher IgG titer in their sera, unlike the IgA level that did not show significant changes, neither in the sera nor in the intestine. Although i.d. route of immunization can induce mucosal immune responses [19, 44–48], in the current study lack of stimulating mucosal immunity could be due to the type of immunization [39–43]. In the contrary to increasing the IgA level in serum, this trend was not observed in MM and NM intestines. Considering the results of survival rates and bacterial detection in the small intestine, it can be concluded that avD3 had no significant effect on an immune response induction against ETEC.

Sera from immunized and sham-immunized groups reacted with similar protein bands of ETEC and OMV. It seems that under natural conditions there are some antibodies against *E.coli* (as normal gut microbiota), which their levels goes up during immunization. We further analyzed the presence of anti- K99 specific antibodies in immune sera. Fimbria K99 is a highly protective antigen against ETEC infection in cattle and mice [14, 46]. Comparing purified K99 peptide along with ETEC K99+ -derived OMVs and whole germ shows that OMVs is not able to induce an antibody response against K99 (Fig. 3). This defect in the immune response is probably due to the removal of fimbria fractions during OMV purification. Nevertheless, our results indicated that anti-K99 antibodies may not be required to induce protective immunity.

Survival rates disclosed a partial protection after challenging with 10^3^ CFU bacteria, while a complete immunity was shown in the challenge with lower dose (10^2^ CFU) bacteria. Partial immunity may be due to the incomplete transmission of antibody from mother to neonates. Since the levels of specific antibodies (IgG and IgA) in neonates were similar to dams, it could be speculated that maternal derived antibodies are effective but not adequate for 10^3^ CFU of ETEC. This is noteworthy that under natural conditions, non-immunized animals have low levels of antibody to ETEC and the protection mainly deals with the specificity and adequate level of antibodies. Results of bacterial detection in the small intestine can provide a further evidence for the above statement. However, the distinct specificity of antibodies and their protection level remains to be elucidated.

## Conclusions

In this report ETEC outer membrane vesicles was prepared and tested alone or in formulation with avD3 as a new candidate for colibacillosis vaccine. Immunization of dam mice induced serum and mucosal antibody responses and could elicit protection in neonates. Furthermore, infant mice born to immunized dam had higher survival rates at post challenge by lower dose bacteria. Vitamin D3 did not indicate a positive effect on mucosal and systemic immune responses. Our study may also contribute to the development of those vaccinology methods, which trigger mucosal immunity and result in passive immunity in offspring.

## Research limitation

- Report demonstrated that the use of vitamin D3 along with ETEC-derived OMVs does not increase the IgG and IgA levels production.
- Vitamin D3 had no significant effect on neonatal protection against ETEC lethality.
- Report highlighted that transferring passive immunity to neonate, at sensitive time after the birth, needs more investigation on stimulating the mucosal immunity.

## Abbreviations

ETEC: enterotoxigenic *Escherichia coli*
OMV: outer membrane vesicles
avD3: active form of vitamin D3
i.d.: intradermal route
MM: mother mice
NM: neonatal mice
